# Effects of Branched-Chain Amino Acids on the Inflammatory Response Induced by LPS in Caco-2 Cells

**DOI:** 10.3390/metabo14010076

**Published:** 2024-01-22

**Authors:** Bruna Ruschel Ewald Vega Garcia, Edson Naoto Makiyama, Geni Rodrigues Sampaio, Rosana Aparecida Manólio Soares-Freitas, Andrea Bonvini, Andressa Godoy Amaral, Silvana Bordin, Ricardo Ambrósio Fock, Marcelo Macedo Rogero

**Affiliations:** 1 Department of Nutrition, School of Public Health, University of São Paulo, São Paulo 01246-904, Brazil; ruschel@usp.br (B.R.E.V.G.); genirs@usp.br (G.R.S.); rosanaso@usp.br (R.A.M.S.-F.); 2 Department of Clinical and Toxicological Analysis, School of Pharmaceutical Sciences, University of São Paulo, São Paulo 05508-000, Brazil; edson.makiyama@usp.br (E.N.M.); hemato@usp.br (R.A.F.); 3 Department of Food and Experimental Nutrition, Faculty of Pharmaceutical Sciences, University of Sao Paulo, São Paulo 05508-000, Brazil; abonvini@alumni.usp.br; 4 Department of Physiology and Biophysics, Institute of Biomedical Sciences, University of Sao Paulo, São Paulo 05508-000, Brazil; andressa_amaral@usp.br (A.G.A.); sbordin@icb.usp.br (S.B.); 5 Food Research Center (FoRC), CEPID-FAPESP (Research Innovation and Dissemination Centers São Paulo Research Foundation), São Paulo 05508-000, Brazil

**Keywords:** inflammation, branched-chain amino acids, Caco-2 cells, lipopolysaccharide

## Abstract

Branched-chain amino acids (BCAA) are essential for maintaining intestinal mucosal integrity. However, only a few studies have explored the role of BCAA in the modulation of intestinal inflammation. In this study, we investigated in vitro effects of BCAA on the inflammatory response induced by lipopolysaccharide (LPS) (1 µg/mL) in Caco-2 cells. Caco-2 cells were assigned to six groups: control without BCAA (CTL0), normal BCAA (CTL; 0.8 mM leucine, 0.8 mM isoleucine, and 0.8 mM valine); leucine (LEU; 2 mM leucine), isoleucine (ISO; 2 mM isoleucine), valine (VAL; 2 mM valine), and high BCAA (LIV; 2 mM leucine, 2 mM isoleucine, and 2 mM valine). BCAA was added to the culture medium 24 h before LPS stimulation. Our results indicated that BCAA supplementation did not impair cell viability. The amino acids leucine and isoleucine attenuated the synthesis of IL-8 and JNK and NF-kB phosphorylation induced by LPS. Furthermore, neither BCAA supplementation nor LPS treatment modulated the activity of glutathione peroxidase or the intracellular reduced glutathione/oxidized glutathione ratio. Therefore, leucine and isoleucine exert anti-inflammatory effects in Caco-2 cells exposed to LPS by modulating JNK and NF-kB phosphorylation and IL-8 production. Further in vivo studies are required to validate these findings and gather valuable information for potential therapeutic or dietary interventions.

## 1. Introduction

In healthy humans, the intake of nine amino acids is essential because they cannot be synthesized endogenously. The branched-chain amino acids (BCAA) leucine, valine, and isoleucine belong to this group [1,2]. Most dietary proteins consist of 20% BCAA. They represent approximately 35% of the indispensable amino acid requirements of mammals [1,3].

BCAA exerts potential therapeutic effects by mitigating the loss of lean mass during body mass reduction, promoting the healing process, and enhancing muscle protein balance in elderly individuals [4,5]. Among the BCAA, leucine plays a prominent role in stimulating protein synthesis, as an increase in the intracellular concentration of this amino acid promotes the activation of the mammalian target of rapamycin (mTOR), which is involved in activating protein synthesis and inhibiting protein degradation [4,6].

The gastrointestinal tract is an important source of inflammatory mediators that play key roles in endotoxemia and the metabolic response to sepsis [7]. The production of inflammatory modulators is controlled by several signaling pathways, including the nuclear factor-kappa B (NF-κB), Janus kinase/signal transducer and activator of transcription (JAK-STAT), and mitogen-activated protein kinase (MAPK) pathways [8]. NF-κB is a ubiquitous transcription factor that is particularly important for the transcriptional control of genes that participate in the response to sepsis and inflammation [9]. NF-κB is activated in different cell types by many stimulants, including viral and bacterial pathogens, and pro-inflammatory cytokines, such as interleukin (IL)-1, IL-6, IL-8, IL-12, and tumor necrosis factor (TNF)-α [10]. In this context, IL-8 plays a major role in pro-inflammatory signaling, as it is elevated in patients with Crohn’s disease or ulcerative colitis, and its levels correlate with intestinal inflammation [11]. Furthermore, the c-Jun N-terminal kinase (JNK) pathway represents a subgroup of the MAPK pathway, which plays an important role in various inflammatory disease states, including inflammatory bowel disease [12].

Dietary amino acids act as essential precursors for the synthesis of glutathione, nitric oxide, polyamines, purine, and pyrimidine nucleotides and amino acids (alanine, citrulline, and proline) in the small intestinal mucosa and play a role in intestinal mucosal mass and integrity [13]. Notably, the small intestinal mucosa contains significant amounts of BCAA transaminase and branched-chain alpha-ketoacid dehydrogenase and is therefore highly proficient in BCAA catabolism [14]. Furthermore, BCAA can improve intestinal morphology and cell proliferation, increase intestinal amino acid absorption by mediating the expression of intestinal amino acid transporters, and promote intestinal protein turnover [15].

Previous studies conducted by our group indicated that BCAA can modulate the inflammatory response in various cell types, such as macrophages [16] and mesenchymal stem cells [17]. Bonvini et al. [16] investigated the effects of different BCAA supplementation protocols on the inflammatory response of lipopolysaccharide (LPS)-stimulated RAW 264.7 macrophages. BCAA significantly increased cell viability and IL-10 synthesis, while TNF-α and prostaglandin E2 synthesis were not altered. Sartori et al. [17] evaluated the effect of BCAAs on the immunomodulatory properties of mesenchymal stem cells (MSC). Supplementation with BCAA increases MSC proliferation and metabolic activity. Further, BCAA supplementation also altered the immunomodulatory capacity of MSC by decreasing the p-NFкB/NFкB and increasing the synthesis of the anti-inflammatory mediators transforming growth factor beta and prostaglandin E_2_. Furthermore, in vivo studies have demonstrated the effects of the amino acid leucine on intestinal inflammatory response. Liu et al. [18] verified that leucine could alleviate LPS-induced inflammatory responses by down-regulating the NF-κB signaling pathway and evoking the mTOR/p70S6K signaling pathway, which may be involved in the regulation of the intestinal immune system in chicken embryos. However, the role of BCAA in modulating inflammatory responses in intestinal cells remains unclear. In this context, the current study aimed to investigate the effect of BCAA on the LPS-induced inflammatory response in human adenocarcinoma cells (Caco-2).

## 2. Materials and Methods

### 2.1. Caco-2 Cells Culture

Caco-2 cells were obtained from the Adolfo Lutz Institute (catalog number CCIAL-064) and cultured in growth medium (Dulbecco’s modified Eagle’s medium (DMEM) containing 10% fetal bovine serum, 4 mM glutamine, and 50 mg/L gentamicin sulfate as an antibiotic). The cell culture medium was obtained from VitroCell (Campinas, São Paulo, Brazil). The experimental groups were derived from this culture medium by supplementation with different concentrations of BCAA.

The number of passages of each cell line ranged from 50 to 70. The cell culture flasks had an area of 75 cm^2^ (Nest Scientific, Phoenix, AZ, USA). Subculturing was performed when the cells reached at least 80% confluence in a flask using a 0.25% trypsin-EDTA solution (Gibco^TM^, Life Technologies Corporation, Grand Island, NY, USA). At this stage, the cells were resuspended in the culture medium and seeded in 12-well plates with 5 × 10^4^ cells per well in a final volume of 1 mL. The cells were maintained in an incubator at 37 °C, with an atmosphere of 5% CO_2_ and 90% relative humidity, for a period of 21 days, with medium changes three times a week. 

### 2.2. Treatment Protocol

After the cell growth and cell culture stage, the culture medium was removed, and the 5 × 10^4^ cells were assigned to six different experimental groups: CTL0 (DMEM without BCAA), CTL (DMEM containing 0.8 mmol/L of each BCAA), LEU (DMEM containing 2 mmol/L of leucine, 0.8 mmol/L of isoleucine and 0.8 mmol/L of valine), ISO (DMEM containing 2 mmol/L of isoleucine, 0.8 mmol/L of leucine and 0.8 mmol/L of valine), VAL (DMEM containing 2 mmol/L of valine, 0.8 mmol/L of isoleucine and 0.8 mmol/L of leucine), and LIV (DMEM containing 2 mmol/L of each BCAA). We used 0.8 mmol/L BCAA, present in DMEM with 10% fetal bovine serum, to represent the physiological situation [19]. A final concentration of 2 mM of each BCAA in the supplemented groups was selected because data from the literature have already demonstrated that higher concentrations can promote increased oxidative stress, increased inflammatory effects, and, consequently, cell death [20]. 

The cells were then treated with culture medium (1 mL/well) for 24 h. Subsequently, cells in every group were either stimulated with 1 µg/mL of LPS from *Escherichia coli*, serotype 055:B5 (Sigma Chemical Company, Saint Louis, MO, USA) for 30 min (western blotting assay) or 24 h (cell viability assay, cytokine measurement in the cell supernatant, glutathione peroxidase activity, and intracellular reduced glutathione (GSH)/oxidized glutathione (GSSG) ratio. The chosen dose of LPS was based on a study [21] that demonstrated that 1 μg/mL of LPS induces the production of IL-8 from Caco-2 cells, and this dose does not affect Caco-2 cell viability [22].

### 2.3. MTT Assay 

The 3-[4,5-dimethylthiazol-2-yl]-2,5-diphenyltetrazolium bromide (MTT) assay is based on the colorimetric determination of blue formazan crystals formed by reduced MTT in the culture medium. The number of formazan crystals was directly proportional to the number of viable cells [23]. After treatment with BCAA and stimulation with LPS, the culture medium was replaced by CTL culture medium (900 µL/well) and added with 100 μL of MTT solution (5 mg/mL in PBS). Incubation lasted for 3 h at 37 °C in an atmosphere containing 5% CO_2_. The medium was then removed and 1 mL of dimethyl sulfoxide was added to each well to dissolve the formazan crystals. The plates were kept at room temperature, protected from light, and agitated for 30 min. The light absorbance at 570 nm was measured with a spectrophotometer (EL800 Universal Microplate Reader Bio-Tek^®^ Instruments, Winooski, VT, USA) using a reference wavelength of 650 nm.

### 2.4. Intracellular Concentrations of Reduced and Oxidized Glutathione

The concentrations of total glutathione (GSH + GSSG) and GSH were assessed using a colorimetric assay (GSH + GSSG/GSH Assay Kit; Abcam, ab239709, Cambridge, MA, USA) following the manufacturer’s instructions. In this assay, cells were collected by centrifugation at 700× *g* for 5 min at 4 °C. Supernatant was removed and the cell pellet was resuspended in 0.5 mL ice-cold phosphate-buffered saline and centrifuged at 700× *g* for 5 min at 4 °C. Later, the supernatant was removed and cells were lysed in 80 μL ice-cold Glutathione Buffer and incubated on ice for 10 min. Subsequently, 20 μL of 5-sulfosalicylic acid (5%) was added and the samples were centrifuged at 8000× *g* for 10 min. The supernatant was transferred to a tube and used for the glutathione assay. The concentration of GSSG was determined by calculating the difference between the total glutathione and GSH values.

### 2.5. Glutathione Peroxidase (GPX) Enzyme Activity 

GPx activity was assessed using a colorimetric assay kit (ab102530, Abcam, Cambridge, MA, USA) following the manufacturer’s instructions. In this assay, cells were washed with cold phosphate-buffered saline and resuspended in 200 μL of cold Assay Buffer. The samples were homogenized quickly by pipetting up and down a few times on ice. Cells were centrifuged at 4 °C at 10,000× *g* for 15 min using a cold microcentrifuge to remove any insoluble material. The supernatant was collected, transferred to a clean tube, and stored on ice before starting the assay. Total protein concentration in the samples was measured using the Pierce™ BCA protein analysis kit (Thermo Fisher Scientific, Waltham, MA, USA) according to the manufacturer’s instructions. The GPx activities were normalized by the protein concentrations.

### 2.6. Cytokine Concentration in Cell Culture Supernatant 

The immunoassay, employing Multiplex microsphere technology (MILLIPLEX^®^ map kit; Cat. No. HCYTOMAG-60K-05), utilizes a specialized platform, the Luminex MAGPIX system (Merck Millipore Corporation, Darmstadt, Germany). This system facilitates the simultaneous quantification of multiple cytokines. In this study, it was employed to measure the concentrations of IL-8, IL-10, and tumor necrosis factor (TNF)-alpha in the cell culture supernatant. All samples were analyzed in duplicate. 

### 2.7. Protein Extraction and Western Blotting

Caco-2 cells were lysed in RIPA^®^ buffer (Sigma-Aldrich, St. Louis, MO, USA) supplemented with phosphatase buffer (1M NaF and 10 mM sodium orthovanadate) and protease inhibitors (22.96 mM PMSF and Protease Inhibitor Cocktail^®^, Sigma-Aldrich, St. Louis, MO, USA). Total protein concentration in the samples was measured using the Pierce™ BCA protein analysis kit (Thermo Fisher Scientific, Waltham, MA, USA) according to the manufacturer’s instructions. The protein extracts were boiled for 10 min in Laemlli buffer in a water bath and maintained at −80 °C for future analysis.

An amount of 30 µg of total proteins was resolved via electrophoresis on 10% TGX ™ FastCast™ polyacrylamide gel (BioRad, Hercules, CA, USA) using a Mini-PROTEAN^®^ mini gel device (BioRad, Hercules, CA, USA). After electrophoresis, the proteins were electrically transferred to nitrocellulose membranes for 10 min in the Power Blotter System^®^ (Life Technologies, Shanghai, China). The membranes were incubated at room temperature for 1 h, under agitation, in TSBT (10 mM Tris, 1.5 mM NaCl, pH 7.6, 0.1% Tween) with 5% BSA to block the nonspecific binding of antibodies to the membranes. Membranes were incubated overnight at 4 °C with primary antibodies against JNK (#3708), pSAPK/JNK (Thr183/Tyr185) (#4668), NFκB p65 (#4764), pNFκB (Ser536) (#3031), AMPKα (#2532), pAMPKα (Thr172) (#2531), mTOR (#2983), p mTOR (Ser2448) (#5536), Tak-1 (# 5206), pTAK-1 (#4508), and β-actin (#8457) (Cell Signaling Technology, Beverly, MA, USA). The antibodies were diluted (1:1000) in TSBT with 3% BSA. Following that, membranes were incubated with a secondary HRP-conjugated antibody (#7074) (Cell Signaling Technology, Beverly, MA, USA) for 60 min at room temperature and then incubated with a solution containing the chemiluminescence reagent Clarity™ Western ECL Substrate (BioRad, Hercules, CA, USA). The intensity of the colored bands was determined via densitometry using ImageQuant™ LAS 4000 (GE Healthcare, Chicago, IL, USA). Band intensities were normalized to β-actin to determine the relative content of the primary targets.

### 2.8. Statistical Analysis 

The results were expressed as mean ± standard error of the mean. All results were subjected to a test of homogeneity of variances (Cochran’s C-test, Hartley test, and Bartlett test). An analysis of variance (ANOVA) was performed, followed by Tukey’s test to identify significant differences. The *t*-student test was applied for related data to evaluate the differences between the control groups and groups supplemented with BCAA (LEU, ISO, VAL, and LIV) and stimulated with LPS. GraphPad Prism software (version 9.3.1) was employed and a significance level of 0.05 was adopted.

## 3. Results

### 3.1. Cell Viability

**Figure 1:**
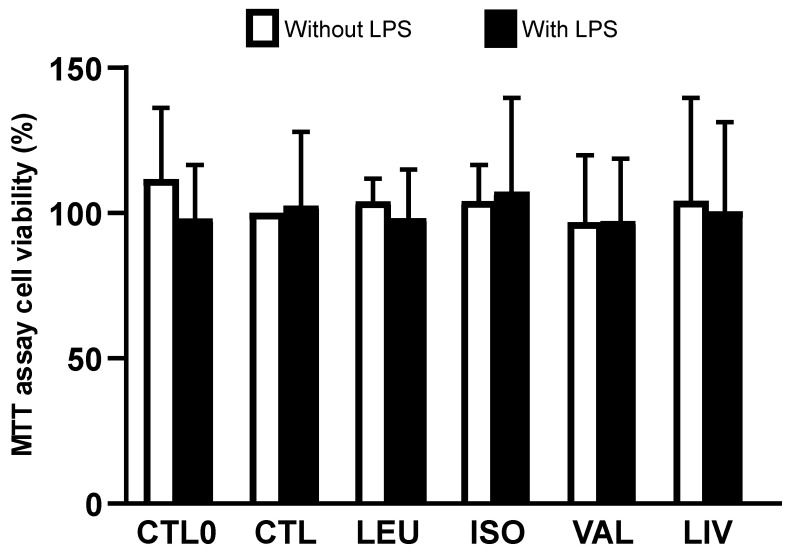
Cell viability. CTL0: negative control without BCAA; CTL: 0.8 mmol/L of each BCAA; LEU: 2 mmol/L of leucine; ISO: 2 mmol/L of isoleucine; VAL: 2 mmol/L of valine; LIV: 2 mmol/L of leucine, 2 mmol/L of isoleucine and 2 mmol/L of valine. Data are presented as mean ± SEM (N = 8/group).

No significant differences in cell viability were observed among the groups irrespective of LPS stimulation (Figure 1). No significant difference was found when only the groups supplemented with BCAA (LEU, ISO, VAL, and LIV) and stimulated with LPS were compared with the CTL0 and CTL groups stimulated with LPS (Figure S4).

### 3.2. Intracellular Concentrations of Reduced and Oxidized Glutathione

**Figure 2:**
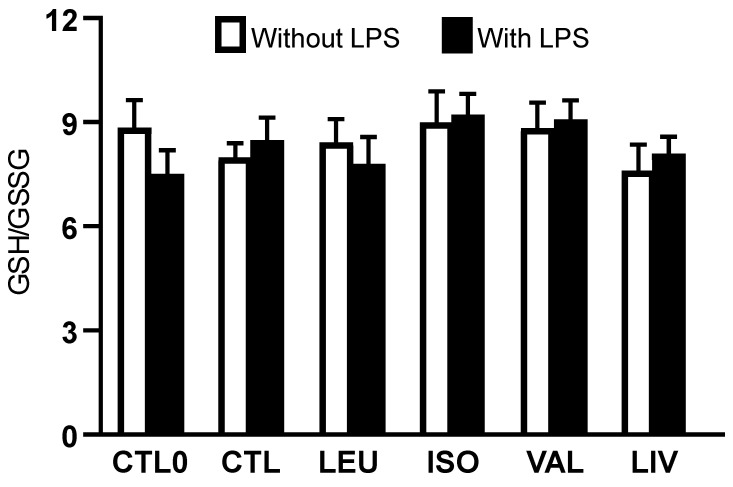
Intracellular reduced glutathione (GSH) to oxidized glutathione (GSSG) ratio. CTL0: negative control without BCAA; CTL: 0.8 mmol/L of each BCAA; LEU: 2 mmol/L of leucine; ISO: 2 mmol/L of isoleucine; VAL: 2 mmol/L of valine; LIV: 2 mmol/L of leucine, 2 mmol/L of isoleucine and 2 mmol/L of valine. Data are presented as mean ± SEM (N = 6/group).

The intracellular GSH/GSSG ratio (Figure 2) did not differ among groups, irrespective of LPS stimulation. No significant difference was found when only the groups supplemented with BCAA (LEU, ISO, VAL, and LIV) and stimulated with LPS were compared with the CTL0 and CTL groups stimulated with LPS (Figure S5).

### 3.3. Activity of Glutathione Peroxidase (GPx)

**Figure 3:**
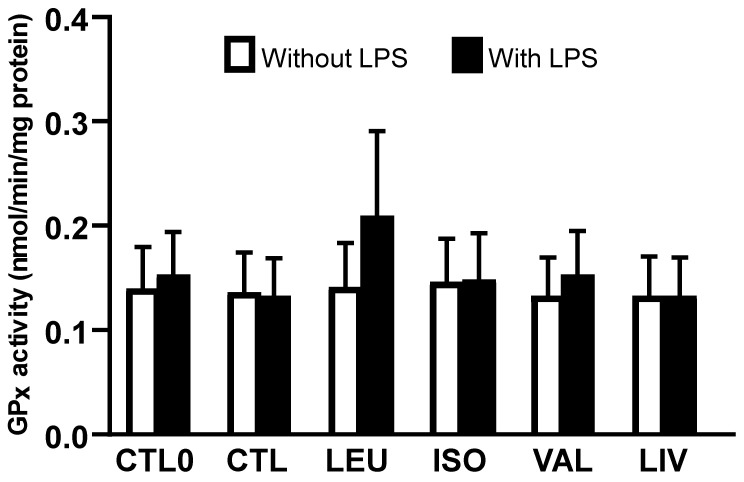
Activity of glutathione peroxidase (GPx) enzyme. CTL0: negative control without BCAA; CTL: 0.8 mmol/L of each BCAA; LEU: 2 mmol/L of leucine; ISO: 2 mmol/L of isoleucine; VAL: 2 mmol/L of valine; LIV: 2 mmol/L of leucine, 2 mmol/L of isoleucine and 2 mmol/L of valine. Data are presented as mean ± SEM (N = 6/group).

The activity of the GPx enzyme (Figure 3) did not differ among the groups, irrespective of LPS stimulation. No significant differences were observed when comparing only the groups supplemented with BCAA (LEU, ISO, VAL, and LIV) and stimulated with LPS to the CTL0 and CTL groups stimulated with LPS (Figure S6).

### 3.4. Cytokine Concentration in Cell Culture Supernatant

**Figure 4:**
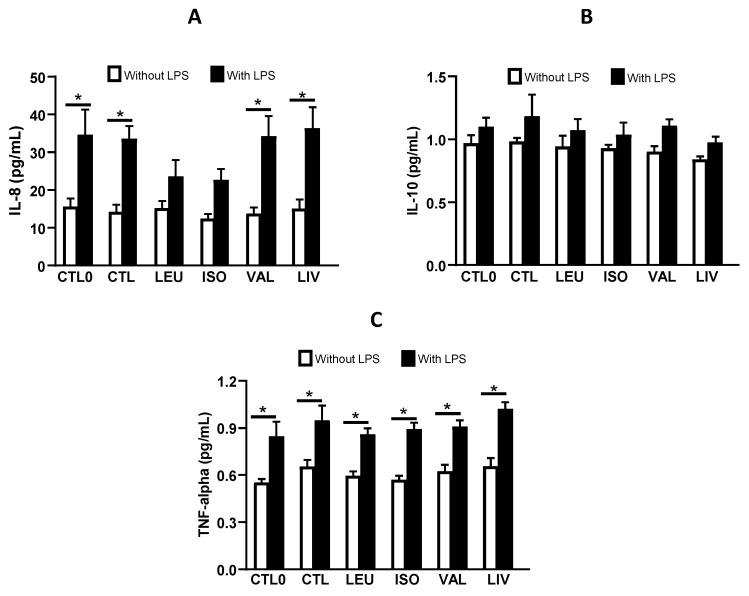
In vitro IL-8 (**A**), IL-10 (**B**), and TNF-alpha (**C**) production. CTL0: negative control without BCAA; CTL: 0.8 mmol/L of each BCAA; LEU: 2 mmol/L of leucine; ISO: 2 mmol/L of isoleucine; VAL: 2 mmol/L of valine; LIV: 2 mmol/L of leucine, 2 mmol/L of isoleucine and 2 mmol/L of valine. * *p* < 0.05. Data are presented as mean ± SEM (N = 6/group).

We found that LPS induced a significant increase in IL-8 production in CTL0, CTL, VAL, and LIV cells compared to their respective non-LPS-stimulated counterparts (*p* < 0.05). In contrast, supplementation with Leu (LEU group) or isoleucine (ISO group) attenuated the increase in IL-8 production induced by LPS (Figure 4A). IL-10 production was similar among all groups, irrespective of the use of LPS (Figure 4B). All groups stimulated with LPS showed higher TNF-alpha production compared to their non-LPS-stimulated counterparts (*p* < 0.05) (Figure 4C). IL-8 production was significantly lower (*p* < 0.05) in the LEU and ISO groups in comparison to the CTL0 and CTL groups stimulated with LPS (Figure S1). No significant differences were observed when comparing only the groups supplemented with BCAA (LEU, ISO, VAL, and LIV) to the CTL0 and CTL groups stimulated with LPS in relation to the IL-10 and TNF-alpha production (Figure S7). 

### 3.5. Content and Phosphorylation of Proteins

**Figure 5:**
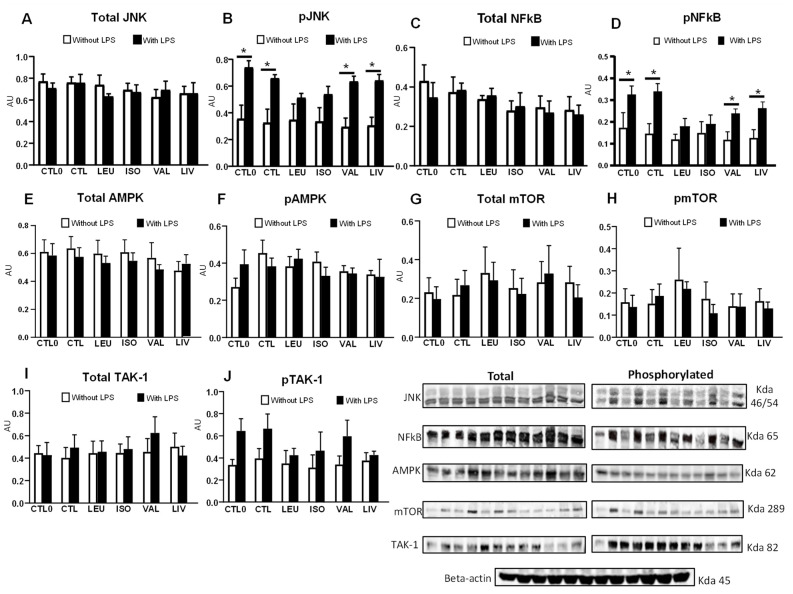
Expression and phosphorylation of the JNK, NFκB, AMPK, mTOR, and TAK-1 in Caco-2 cells. (**A**) Total and (**B**) phosphorylated JNK; (**C**) total and (**D**) phosphorylated NFκB; (**E**) total and (**F**) phosphorylated AMPK; (**G**) total and (**H**) phosphorylated mTOR; and (**I**) total and (**J**) phosphorylated TAK-1. CTL0: negative control without BCAA; CTL: 0.8 mmol/L of each BCAA; LEU: 2 mmol/L of leucine; ISO: 2 mmol/L of isoleucine; VAL: 2 mmol/L of valine; LIV: 2 mmol/L of leucine, 2 mmol/L of isoleucine and 2 mmol/L of valine. * *p* < 0.05. Data are presented as mean ± SEM (N = 5/group). AU, arbitrary units; JNK, c-Jun N-terminal Kinase; NFκB, nuclear factor kappa B p65 subunit; AMPK, AMP-activated protein kinase; mTOR, mechanistic target of rapamycin; TAK1, transforming growth factor-beta-activated kinase 1.

LPS was able to significantly stimulate JNK (Figure 5B) and NF-kB (Figure 5D) phosphorylation in cells belonging to the CTL0, CTL, VAL, and LIV groups (as compared to their non-LPS stimulated counterparts; *p* < 0.05). Cells supplemented with leucine or isoleucine instead did not exhibit significant changes in the phosphorylation of JNK (Figure 5B) and NF-kB (Figure 5B) content upon LPS stimulation. The levels and phosphorylation of AMPK (Figure 5E,F), mTOR (Figure 5G,H), and TAK-1 (Figure 5I,J) proteins were similar among the groups, irrespective of the use of LPS. JNK (Figure S2) and NF-kB (Figure S3) phosphorylation were significantly lower (*p* < 0.05) in the LEU and ISO groups in comparison to the CTL0 and CTL groups stimulated with LPS. No significant differences were observed when comparing only the groups supplemented with BCAA (LEU, ISO, VAL, and LIV) to the CTL0 and CTL groups stimulated with LPS in relation to the AMPK, mTOR, and TAK-1 phosphorylation (Figure S8).

## 4. Discussion

In this study, we explored the potential effects of BCAA on the LPS-induced inflammatory response in Caco-2 cells. This cell line is derived from human colon adenocarcinoma, exhibits enterocyte-like characteristics, and has been widely utilized as an in vitro model to study the molecular mechanisms underlying inflammatory bowel disease [24]. The results of the MTT assay indicated that the selected concentrations of in vitro BCAA supplementation did not affect cell viability. Notably, in our experiments, cell viability exceeded 90% in the supplemented groups (LEU, ISO, VAL, and LIV), indicating that BCAA treatment did not induce cytotoxic effects in Caco-2 cells. Consistent with the findings of this study, De Simone et al. [25] observed that microglial cells supplemented in vitro with BCAA (1 or 10 mM) remained viable for 24 h. A previous study conducted by our group reported no impairment in the viability of stem cells exposed to the same concentrations of BCAA as used in the current experiments [17]. Indeed, in vitro supplementation with BCAA increased cell proliferation. In another study, Bonvini et al. [16] observed that BCAA increases cell viability and modulates cytokine synthesis in RAW 264.7 macrophages.

Lipopolysaccharide (LPS), a major component of the outer membrane of gram-negative bacteria, activates cells through toll-like receptor 4, inducing host cells to produce cytokines and inflammatory mediators [21]. The dose of LPS used in this study (1 µg/mL) did not impair cell viability. Similarly, Fangi et al. [22] found that a similar dose of LPS (1.25 µg/mL), comparable to that used in the present study or even higher (80 µg/mL), does not affect Caco-2 cell viability.

Considering that the balance between cellular antioxidant systems and oxidative stress plays a relevant role in regulating the inflammatory response, we evaluated the antioxidant system of glutathione in Caco-2 cells. This approach was based on determining the intracellular concentrations of GSH and GSSG as well as the activity of glutathione peroxidase. GSH is a tripeptide (gamma-glutamylcysteinylglycine) that acts as a primary non-enzymatic intracellular antioxidant under oxidative stress. GSH, whose concentration ranges from 1 to 10 mM in the intracellular environment, is found in the cytoplasm, mitochondria, and nucleus [26,27,28,29]. In the reactions catalyzed by glutathione peroxidase, two GSH molecules are oxidized to form one GSSG molecule. Once formed, GSSG can be converted back to its reduced form (GSH) through a reaction catalyzed by glutathione reductase. Notably, GSH can regenerate oxidized molecules such as vitamins C and E [30,31]. 

BCAA transaminase and branched-chain α-ketoacid dehydrogenase are present in the intestinal mucosa, providing the enzymatic basis for BCAA catabolism [6]. In this context, we chose to assess the glutathione system because the transamination of BCAA leads to the production of a branched-chain α-ketoacid and glutamate [6,14]. We hypothesized refers to the fact that glutamate could play a role in the synthesis of glutathione since this tripeptide is formed through the action of glutamate cysteine ligase [27]. Nevertheless, the current results obtained in Caco-2 cells showed no effect of BCAA supplementation on the parameters related to the glutathione antioxidant system. This lack of an effect may be related to the fact that glutamate is a truly dispensable amino acid and does not act as a limiting amino acid in glutathione synthesis [30]. Additionally, the metabolism of other amino acids in intestinal cells can generate glutamate, for example, in the reaction catalyzed by the enzyme glutaminase, which produces glutamate and ammonia from glutamine [14]. In addition, BCAA does not have a direct effect on the endogenous synthesis of glutathione, which depends mainly on the amino acids cysteine and glycine [30]. 

The intestinal epithelium serves as a crucial barrier between the luminal environment and the host’s internal milieu. Additionally, it acts as an early signaling system for host immune cells in the gut. Signal transduction pathways that regulate cytokine gene expression are activated in enterocytes in response to different bacteria, toxins, and other classes of antigens [32,33]. IL-8, a pro-inflammatory cytokine synthesized in the intestine, exerts potent chemoattractant action on leukocytes and serves as an indicator of tissue oxidative stress. This cytokine initiates the inflammatory cascade and is an early biomarker of inflammatory response. Increased IL-8 expression contributes to local (intestinal) and distant (other organ) tissue damage [34]. It is noteworthy that activation of the NFκB signaling pathway leads to increased gene expression and secretion of IL-8 by Caco-2 cells. In this context, LPS binding to and subsequent activation of toll-like receptor 4 (TLR4) triggers potent stimulation of the NF-kappa B signaling pathway [35].

In the present study, leucine and isoleucine attenuated the LPS-induced synthesis of IL-8 in Caco-2 cells. Similar effects of leucine and isoleucine were observed by Katayama and Mine [36] in Caco-2 cells pretreated with different amino acids (Cys, Ala, Ile, Leu, Trp, Val, Lys, and His) and then stimulated with hydrogen peroxide (1 mM) to induce oxidative stress. The authors found that the secretion of IL-8 by Caco-2 cells was significantly attenuated by pretreatment with leucine and isoleucine. In this context, there appears to be a potential anti-inflammatory action of leucine and isoleucine in Caco-2 cells, although the mechanisms underlying the attenuation of IL-8 synthesis have not yet been fully elucidated. 

Among the possible mechanisms underlying the effect of the amino acids leucine and isoleucine in the attenuation of IL-8 production, it is relevant to highlight the downregulation of NFκB and the JNK phosphorylation, two proteins that signal the transcription of genes involved in the inflammatory response, including the IL-8 gene [37]. The JNK pathway represents a subgroup of mitogen-activated protein kinases that play a crucial role in various inflammatory disease states, including inflammatory bowel disease. JNK is activated by various environmental stresses and responds to cytokines, such as TNF-α and IL-1. JNK can phosphorylate transcription factors, such as JunB, JunD, c-fos, ATF2, and ATF3. These transcription factors, along with c-Jun, constitute the activator protein-1 transcription factor (AP-1), which regulates the expression of several stress-responsive genes [38]. It is important to note that the promoter region of the IL-8 gene harbors binding sites for the transcription factors NFκB and activator protein-1 (AP-1). These sites are close to each other and to the coding region of the gene [37]. 

We also investigated the content and phosphorylation of adenosine monophosphate (AMP)-activated protein kinase (AMPK), a serine/threonine kinase, and mTOR in Caco-2 cells, considering the role of these proteins in the inflammatory response in intestinal cells. This study aimed to verify the potential additional mechanisms associated with the anti-inflammatory effects of the amino acids leucine and isoleucine in attenuating the synthesis of IL-8 in vitro. AMPK activation has been shown to suppress various pro-inflammatory signaling cascades, including the pro-inflammatory JNK and NFκB pathways. Furthermore, AMPK has been reported to have anti-inflammatory actions that are both dependent and independent of its metabolic effects [39,40,41,42,43,44]. In contrast, the mTOR pathway plays a crucial role in the inflammatory process and inflammatory diseases, primarily by exerting influence on inflammatory mediators. mTOR is a downstream molecule of the PI3K/AKT/mTOR signaling pathway, which plays a key role in cellular transduction and biological processes [45]. It is worth noting that TLR4 activation induced by LPS triggers the activation of the P38MAPK, which, in turn, promotes the activation of mTORC1 that sequentially activates the NFκB transcription factor. Activation of the mTOR signaling pathway in intestinal epithelial cells induces events leading to inflammatory bowel diseases. In addition, activation of the mTOR/NF-κB pathway upregulates IL-8 [46,47]. 

Nevertheless, in the present study, in vitro supplementation with leucine and isoleucine did not significantly modulate the phosphorylation of AMPK and mTOR. Among the possible causes related to the lack of effect of BCAA on AMPK protein phosphorylation, it stands out that this protein, which acts as an energy sensor, is activated by upstream enzymes when the cellular ratio of AMP to adenosine triphosphate (ATP) is elevated due to nutrient deprivation [48]. In the present assay, none of the interventions involved nutrient deprivation, except for the CTL0 group, in which the cells were cultured for 24 h in the absence of BCAA. However, in this group, an increase in AMPK phosphorylation was not observed compared with the other groups studied.

Among the possible factors related to the lack of effect of BCAA supplementation on mTOR protein levels, the chosen doses of BCAA may not be sufficient to induce an increase in mTOR phosphorylation. In this regard, in peripheral blood mononuclear cells exposed to increasing concentrations of BCAA (0.2–12 mmol/L), a significant increase in phosphorylation at Ser2448 (specific to mTORC1) was observed at concentrations of 8, 10, and 12 mmol/L compared to the control group [49]. In this study [49], a high dose of BCAA (10 mmol/L) augmented the phosphorylation of the p65 component of NF-κB, as well as its nuclear translocation. 

Given these results, we chose to evaluate the TAK-1 protein, since this protein can activate both NFκB and JNK [50]. However, our results indicated that the amino acids leucine and isoleucine did not affect TAK-1 phosphorylation. These findings imply that pre-incubation with BCAA for 24 h before LPS stimulation may influence other proteins within the signaling pathway of the TLR4. Consequently, this modulation may contribute to the attenuation of IL-8 synthesis.

This study has several strengths and limitations. Among the strengths, the use of Caco-2 cells is highlighted. These cells are derived from human colon adenocarcinoma cells and share many similarities with absorptive cells of the small intestine [51]. Caco-2 cells are frequently used in in vitro experiments to investigate the intestinal barrier function and inflammatory bowel disease [24]. Another strength of the present study was the assessment of different concentrations of BCAA, allowing the investigation of the potential role of each BCAA in modulating the inflammatory response in Caco-2 cells. Among the limitations is the limited number of specific markers of oxidative stress, which could contribute to a better understanding of the results. This is significant because an increase in oxidative stress is an important factor related to the activation of the NF-κB signaling pathway [52].

## 5. Conclusions

In conclusion, our results showed that leucine and isoleucine display anti-inflammatory effects in Caco-2 cells exposed to LPS by modulating JNK and NF-kB phosphorylation and IL-8 production. These data suggest that leucine and isoleucine may be able to modulate the inflammatory responses in the gut. Therefore, it is essential to validate these findings in vivo in order to gather valuable information for potential therapeutic and dietary interventions.

## Data Availability

The data presented in this study are available in article or Supplementary Material.

## Supplementary Materials

The following supporting information can be downloaded at: https://www.mdpi.com/article/10.3390/metabo14010076/s1. Figure S1. In vitro IL-8 production in Caco-2 cells exposed to LPS; Figure S2. Phosphorylation of the JNK in Caco-2 cells exposed to LPS; Figure S3. Phosphorylation of the NFκB in Caco-2 cells exposed to LPS; Figure S4. Cell viability in Caco-2 cells exposed to LPS; Figure S5. Intracellular reduced glutathione (GSH) to oxidized glutathione (GSSG) ratio in Caco-2 cells exposed to LPS; Figure S6. Activity of glutathione peroxidase (GPx) enzyme in Caco-2 cells exposed to LPS; Figure S7. In vitro IL-10 and TNF-alpha production in Caco-2 cells exposed to LPS; Figure S8. Phosphorylation of the AMPK, mTOR, and TAK-1 in Caco-2 cells exposed to LPS.
