# Supplementary material for: Effects of Branched-Chain Amino Acids on the Inflammatory Response Induced by LPS in Caco-2 Cells
Source: Metabolites. 2024 Jan 22;14(1):76. doi: 10.3390/metabo14010076 (PMC10821323; doi:10.3390/metabo14010076)
Supplement: Supplementary file 1 [file metabolites-14-00076-s001.zip › metabolites-2802224-supplementary.pdf]

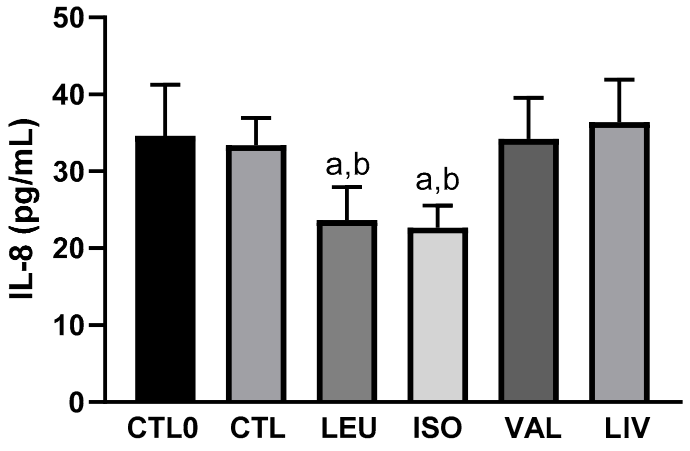

Figure S1. In vitro IL-8 production in Caco-2 cells exposed to LPS. [a] Significant difference ($p < 0.05$) to the CTL0 group; [b] Significant difference ($p < 0.05$) to the CTL group.

CTL0: negative control without BCAA; CTL: 0.8 mmol/L of each BCAA; LEU: 2 mmol/L of leucine; ISO: 2 mmol/L of isoleucine; VAL: 2 mmol/L of valine; LIV: 2 mmol/L of leucine, 2 mmol/L of isoleucine and 2 mmol/L of valine. Data are presented as mean ± SEM (N=6/group).

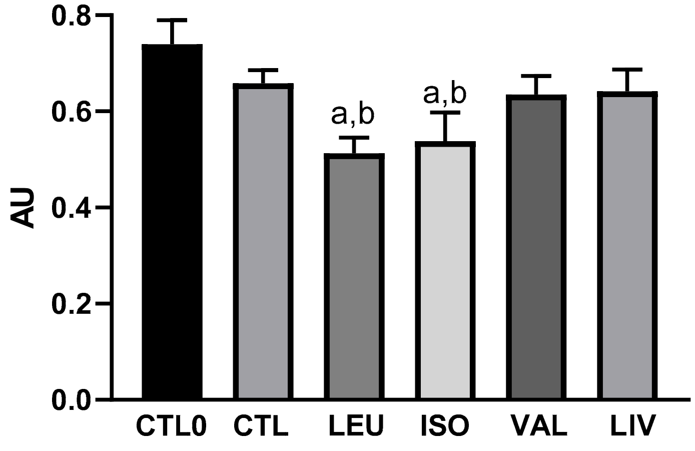

Figure S2. Phosphorylation of the JNK in Caco-2 cells exposed to LPS. [a] Significant difference ($p < 0.05$) to the CTL0 group; [b] Significant difference ($p < 0.05$) to the CTL group.

CTL0: negative control without BCAA; CTL: 0.8 mmol/L of each BCAA; LEU: 2 mmol/L of leucine; ISO: 2 mmol/L of isoleucine; VAL: 2 mmol/L of valine; LIV: 2 mmol/L of leucine, 2 mmol/L of isoleucine and 2 mmol/L of valine. Data are presented as mean ± SEM (N=5/group). AU, arbitrary units; JNK, c-Jun N-terminal Kinase.

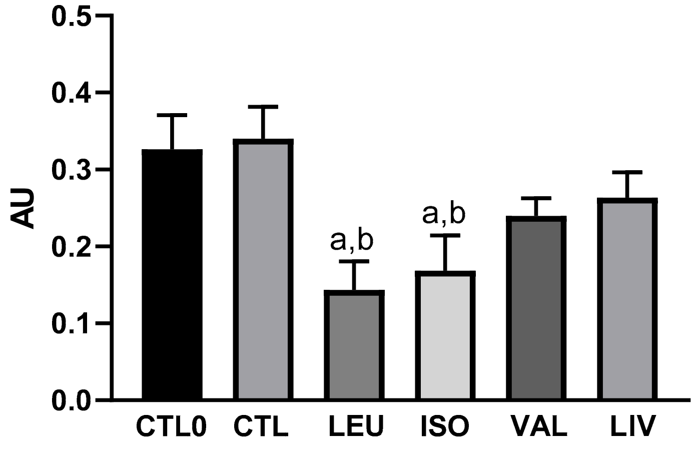

Figure S3. Phosphorylation of the NFκB in Caco-2 cells exposed to LPS. [a] Significant difference ($p < 0.05$) to the CTL0 group; [b] Significant difference ($p < 0.05$) to the CTL group.

CTL0: negative control without BCAA; CTL: 0.8 mmol/L of each BCAA; LEU: 2 mmol/L of leucine; ISO: 2 mmol/L of isoleucine; VAL: 2 mmol/L of valine; LIV: 2 mmol/L of leucine, 2 mmol/L of isoleucine and 2 mmol/L of valine. Data are presented as mean ± SEM (N=5/group). AU, arbitrary units; NFκB, nuclear factor kappa B p65 subunit.

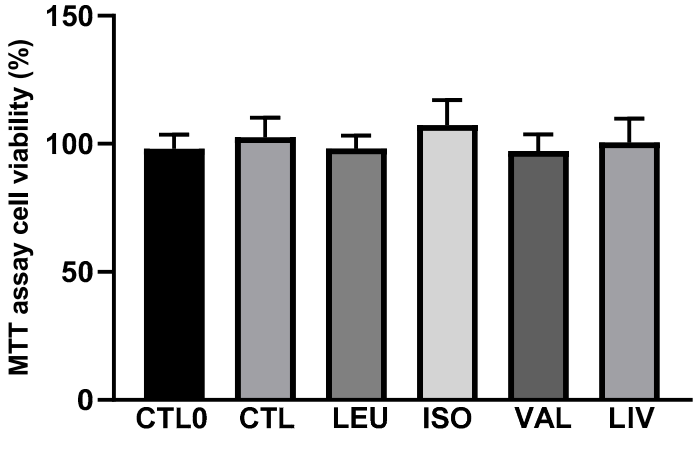

Figure S4. Cell viability in Caco-2 cells exposed to LPS.

CTL0: negative control without BCAA; CTL: 0.8 mmol/L of each BCAA; LEU: 2 mmol/L of leucine; ISO: 2 mmol/L of isoleucine; VAL: 2 mmol/L of valine; LIV: 2 mmol/L of leucine, 2 mmol/L of isoleucine and 2 mmol/L of valine. Data are presented as mean ± SEM (N=8/group).

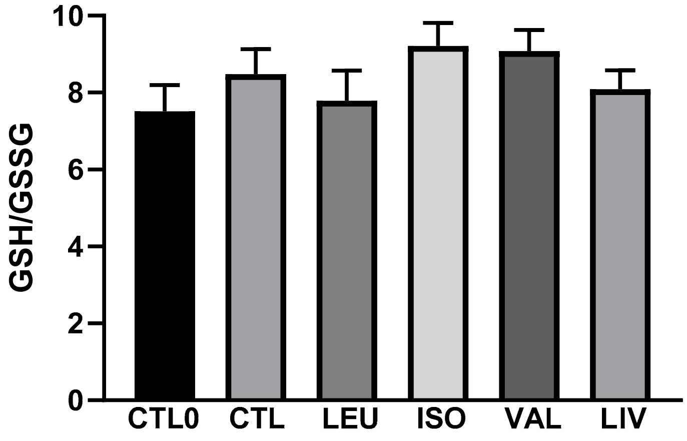

Figure S5. Intracellular reduced glutathione (GSH) to oxidized glutathione (GSSG) ratio in Caco-2 cells exposed to LPS.

CTL0: negative control without BCAA; CTL: 0.8 mmol/L of each BCAA; LEU: 2 mmol/L of leucine; ISO: 2 mmol/L of isoleucine; VAL: 2 mmol/L of valine; LIV: 2 mmol/L of leucine, 2 mmol/L of isoleucine and 2 mmol/L of valine. Data are presented as mean ± SEM (N=6/group).

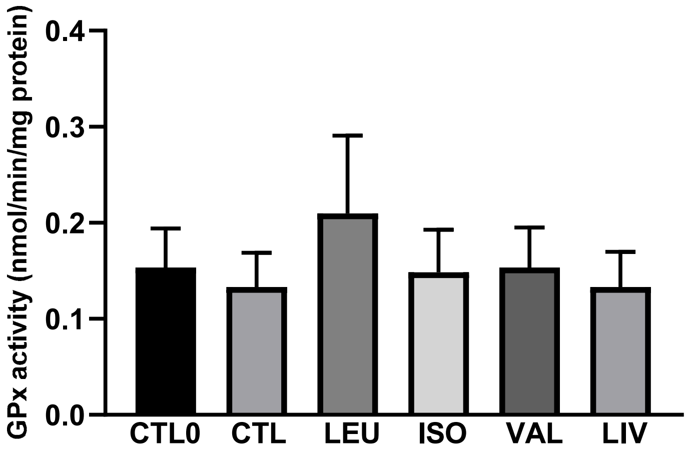

Figure S6. Activity of glutathione peroxidase (GPx) enzyme in Caco-2 cells exposed to LPS.

CTL0: negative control without BCAA; CTL: 0.8 mmol/L of each BCAA; LEU: 2 mmol/L of leucine; ISO: 2 mmol/L of isoleucine; VAL: 2 mmol/L of valine; LIV: 2 mmol/L of leucine, 2 mmol/L of isoleucine and 2 mmol/L of valine. Data are presented as mean ± SEM (N=6/group).

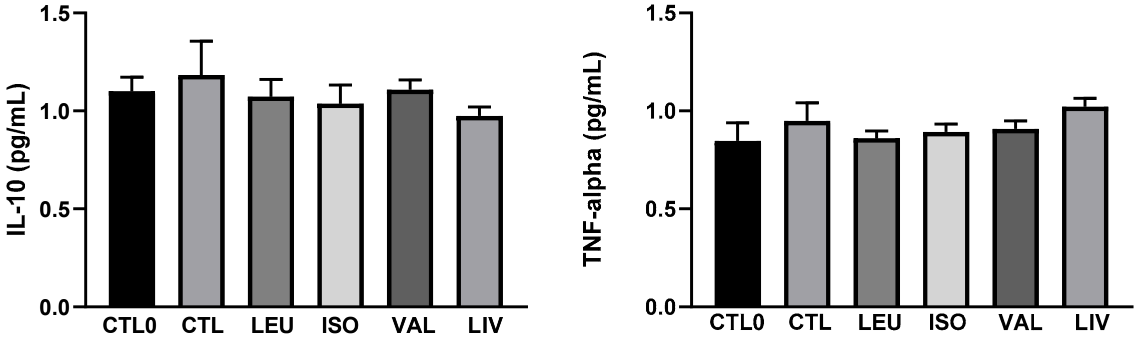

Figure S7. In vitro IL-10 and TNF-alpha production in Caco-2 cells exposed to LPS.

CTL0: negative control without BCAA; CTL: 0.8 mmol/L of each BCAA; LEU: 2 mmol/L of leucine; ISO: 2 mmol/L of isoleucine; VAL: 2 mmol/L of valine; LIV: 2 mmol/L of leucine, 2 mmol/L of isoleucine and 2 mmol/L of valine. Data are presented as mean ± SEM (N=6/group).

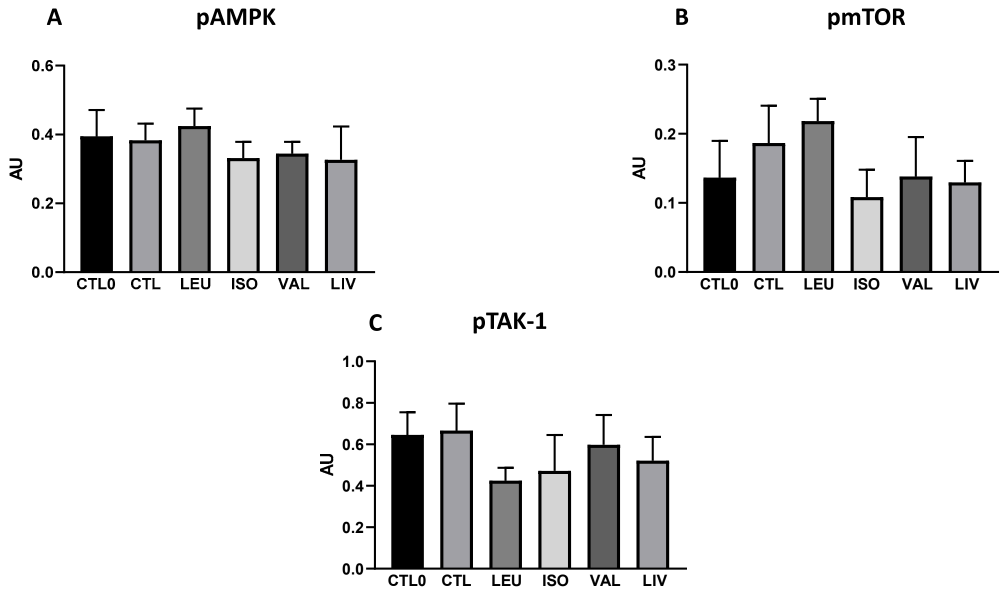

Figure S8. Phosphorylation of the AMPK, mTOR, and TAK-1 in Caco-2 cells exposed to LPS.

(A) phosphorylated AMPK; (B) phosphorylated mTOR; and (C) phosphorylated TAK-1.

CTL0: negative control without BCAA; CTL: 0.8 mmol/L of each BCAA; LEU: 2 mmol/L of leucine; ISO: 2 mmol/L of isoleucine; VAL: 2 mmol/L of valine; LIV: 2 mmol/L of leucine, 2 mmol/L of isoleucine and 2 mmol/L of valine. Data are presented as mean ± SEM (N=5/group). AU, arbitrary units; AMPK, AMP-activated protein kinase; mTOR, mechanistic target of rapamycin; TAK1, transforming growth factor-beta-activated kinase 1.
